# Tumour Size and T-Stage in Pancreatic Cancer Resection Specimens Depend on the Pathology Examination Approach

**DOI:** 10.3390/cancers14102471

**Published:** 2022-05-17

**Authors:** My Linh Tran, Maia Blomhoff Holm, Caroline Sophie Verbeke

**Affiliations:** 1 Department of Pathology, Faculty of Medicine, University of Oslo, 0318 Oslo, Norway; m.l.tran@studmed.uio.no (M.L.T.); m.b.holm@medisin.uio.no (M.B.H.); 2 Department of Pathology, Oslo University Hospital, 0379 Oslo, Norway

**Keywords:** pancreatic cancer, pathology, tumour size, T-stage

## Abstract

**Simple Summary:**

Tumour size is considered a key oncological feature, as it reflects the primary tumour burden. In pancreatic cancer, tumour size also constitutes the defining criterion for staging of the primary tumour (T-stage), which provides essential information for patient treatment, risk stratification in clinical trials and cancer registries. While measurement of tumour size in pancreatic cancer resection specimens is considered accurate and reproducible, this has not been formally proven. This study investigated whether, and in how far, various approaches to tumour size measurement result in different tumour sizes and consequently different T-stages. The study findings show that tumour size and T-stage are different in a significant proportion of cases, depending on whether (i) the tumour was measured in one or two planes and (ii) the macroscopic tumour size was corroborated microscopically. Hence, a divergence in pathology practice may limit the comparability of tumour size and T-stage between institutions.

**Abstract:**

In the eighth edition of the TNM classification for pancreatic ductal adenocarcinoma (PDAC), stages T1 to T3 are defined by tumour size, size measurement being deemed objective and accurate. This study investigated whether various, currently used approaches to tumour measurement result in different tumour sizes and differences in T-stage assignment. In a series of 315 resected PDAC, tumour sizes were measured as follows: macroscopically in a single or in two perpendicular planes and with or without microscopic corroboration. Comparison of the resulting tumour sizes showed that both macroscopic measurement in two planes and microscopic corroboration gave significantly different results (*p* < 0.001). Compared to the most simple approach (macroscopic measurement in one plane), the comprehensive approach (macroscopic measurement in two planes with microscopic corroboration) resulted in a larger tumour size in 263 (83%) cases (mean absolute size difference: 10 mm; mean relative size change: 36%). T-stage assignment differed in 142 (45%) cases between the simple and comprehensive approach and affected 87%, 38% and 48% of the cases deemed to be stage T1, T2 and T3, respectively. In conclusion, tumour size and T-stage are highly approach-dependent. Consensus on an accurate method is required to ensure comparability of these basic data.

## 1. Introduction

For most solid cancer entities, including pancreatic cancer, the size of the tumour is considered of key oncological importance, as it reflects the primary tumour burden. Moreover, according to the eighth edition of the Tumour, Node, Metastasis (TNM) classification published by the Union for International Cancer Control (UICC) and the American Joint Commission on Cancer (AJCC) [1,2], tumour size is the defining criterion for the T-categories T1–T3. While the definition of categories T1 and T2 remained unchanged (T1: ≤20 mm, T2: >20 mm to ≤40 mm) compared to the previous edition [3,4], invasion outside the pancreas as the defining criterion for T3 was replaced by tumour size exceeding 40 mm. Hence, the eighth edition of the AJCC/UICC TNM classification introduced criteria for T1 to T3 that are exclusively based on tumour size, the underlying assumption being that measurement of the largest tumour size is objective and straightforward and therefore accurate and reproducible.

Because tumour staging is key to both individual patient management, clinical research and cancer registries, tumour size is a core data item in national and international data sets for pancreatic cancer [5,6,7,8,9]. However, in spite of the clinical importance attached to tumour size, (inter-)national recommendations for the pathology reporting of surgical resection specimens with ductal adenocarcinoma of the pancreas provide only limited guidance as to how tumour size should be measured. Most guidelines recommend microscopic corroboration of the largest tumour size that was measured macroscopically [5,6,7,8,9]. The reason for this recommendation is that pancreatic cancer grows in a highly dispersed fashion, such that the exact tumour boundary may not be visible by naked-eye inspection [10]. Furthermore, atrophy of flanking pancreatic parenchyma and fibrosis often blur the macroscopic delineation of the tumour. In recognition of the fact that most cancers do not have a perfect spherical shape, it is important that the *largest* tumour size is recorded. This requires measurement in multiple planes, and current specimen dissection techniques allow for measurement in two planes: the plane in which the specimen is sliced and the plane perpendicular to that.

In practice, most pathologists record the tumour size during specimen grossing by measuring the tumour—usually in two dimensions—on the specimen slice in which the tumour appears at its largest expanse. Some will subsequently check the macroscopic size measurement during microscopic examination. Measurement of a third dimension, in the plane across specimen slices, may be performed by some pathologists for cancers in the pancreatic body or tail, for example in the context of a clinical study, but this approach is usually omitted for cancers located in the head of the pancreas [11].

Given the importance of accurate measurement of the largest tumour size for correct T-staging, this study aimed at comparing the results of the above approaches, that is, two-dimensional versus three-dimensional measurement and macroscopic measurement with or without microscopic corroboration. Furthermore, the study aimed at investigating if, and in how far, different approaches to tumour size measurement affected assignment to T-stage.

## 2. Materials and Methods

### 2.1. Study Cohort

This observational study is based on a cohort of consecutive pancreatic ductal adenocarcinomas, for which data were prospectively collected and stored in a database at the Department of Pathology at Oslo University Hospital. Included were all ductal adenocarcinomas of the pancreas that were surgically resected at Oslo University Hospital, Norway, between 1 January 2015 and 31 December 2020. Excluded were tumours that had been treated neoadjuvantly, pancreatic ductal adenocarcinoma that had developed from intraductal papillary mucinous neoplasia and any other tumour entities in the pancreas, including mixed tumour entities, such as mixed neuroendocrine–non-neuroendocrine neoplasms.

### 2.2. Data Collection

For each case in the study cohort, the following data were recorded: type of surgical resection, tumour site (head or body/tail of pancreas), T- and N-stage (according to the 8th edition of the AJCC/UICC TNM classification), presence or absence of lymphatic, vascular and perineural invasion, and resection margin status. Regarding the tumour dimensions, two sets of sizes were extracted from the database: (1) the maximum tumour size measured macroscopically in one specimen slice and the maximum tumour size measured across specimen slices and (2) the same tumour dimensions corroborated by microscopic measurement.

### 2.3. Pathology Examination

**Figure 1:**
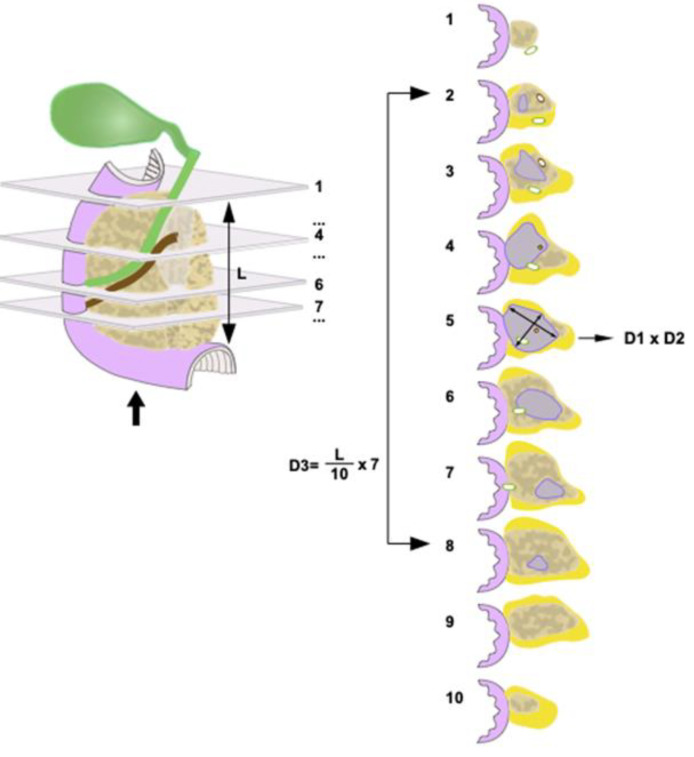
Tumour size measurement in pancreatoduodenectomy specimens. Following specimen slicing in the axial plane, the maximum tumour dimension (D1) is measured on the specimen slice where the tumour is largest. A second tumour dimension can be measured on the same slice perpendicular to the first measurement (D2); this size is not considered in this study, as it is by definition not the largest. The dimension measured in the plane perpendicular to the axial plane (D3) is calculated based on the craniocaudal length of the pancreatic head (L), the total number of axial specimen slices and the number of tumour-bearing slices. In case the tumour was visible on only one side of either the first or the last specimen slice, half of the slice thickness was considered in the calculation. To ensure correct identification of the cranial and caudal ends of the tumour (macroscopically assumed in specimen slices 2 and 8), both adjacent slices (i.e., slices 1 and 9) were also embedded.

*Grossing* of all specimens, undertaken by a pancreatic pathologist (CSV) or by residents and trained technical staff under the supervision of the pancreatic pathologist, was carried out in accordance with national guidelines [12] and a rigorous departmental standard operating procedure, as described previously [13,14]. Briefly, pancreatoduodenectomy specimens were dissected by axial slicing, yielding for each case between 10–15 slices. During macroscopic examination, the largest tumour size was routinely measured in the plane of specimen slicing and the plane perpendicular to that, as follows (Figure 1).

This (third) tumour size is hereafter referred to as “3D-macro”.

Once the specimen slices were laid out in sequential order, the specimen slice in which the tumour was at its largest expanse was identified. The largest tumour dimension was measured on that slice (hereafter referred to as “2D-macro”). The second tumour dimension, which was measured on the same specimen slice, perpendicularly to the largest dimension, was not considered in the current study, as it did not represent the largest tumour size in that plane. Furthermore, the number of the axial specimen slices that were involved by tumour was recorded. The tumour dimension in the craniocaudal plane was then calculated based on the length of pancreatic head in craniocaudal direction (measured before axial slicing), the total number of specimen slices and the number of tumour-bearing slices, according to the following formula:


$$\left(\frac{\mathrm{craniocaudal}\;\mathrm{length}\;\mathrm{of}\;\mathrm{pancreas}}{\mathrm{total}\;\mathrm{number}\;\mathrm{of}\;\mathrm{axial}\;\mathrm{slices}}\right)\times \;\mathrm{number}\;\mathrm{of}\;\mathrm{axial}\;\mathrm{slices}\;\mathrm{with}\;\mathrm{tumour}.$$

With this approach, the accuracy of the measurement in the craniocaudal direction was determined by the thickness of the specimen slices. In some cases, tumour tissue was visible only on one side but not on both sides of the specimen slice(s) containing the edge of the tumour. To increase accuracy on such occasions, only half of the slice thickness for that particular slice (or for both slices) was used in the size calculation. Given an average slice thickness of 3 mm, the accuracy of size measurement across specimen slices was 1.5 mm. 

**Figure 2:**
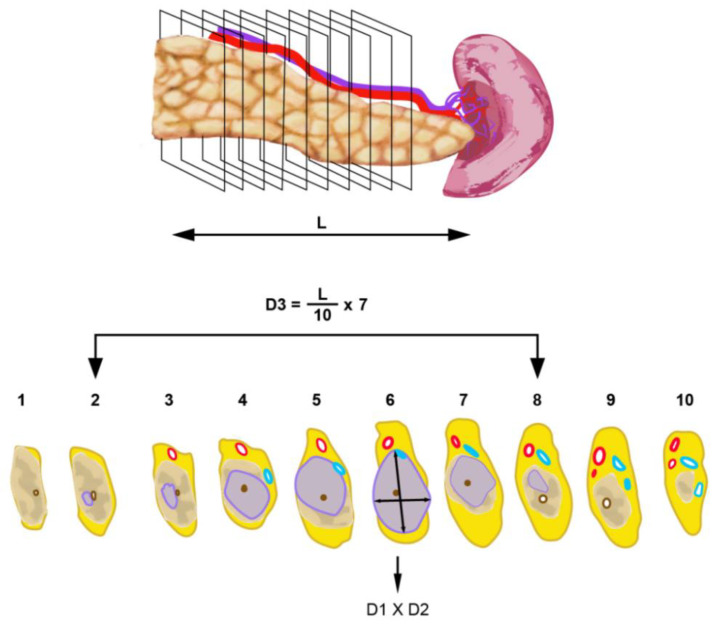
Tumour size measurement in distal pancreatectomy specimens. Following specimen slicing in the sagittal plane, the maximum tumour dimensions (D1 and D3) are measured in a similar fashion as described in Figure 1.

Distal pancreatectomy specimens were dissected by serial slicing in the sagittal plane, that is, perpendicular to the longitudinal axis of the pancreatic body and tail. A similar procedure was followed to evaluate the maximum tumour size in three dimensions: by measuring the maximum tumour size on the slice with the largest tumour expanse (2D-macro) and by calculating the tumour size across sagittal specimen slices (3D-macro), as illustrated in Figure 2.

Total pancreatectomy specimens were divided by the pathologist into a “pancreatoduodenectomy” and a “distal pancreatectomy” part, in which the tumour dimensions were measured as described above. 

To allow review of the macroscopic findings, each case was photo-documented by an overview image of all specimen slices as well as close-up images of individual slices.

*Tissue sampling* was extensive in each case and included complete embedding of (i) the specimen slice deemed to contain the largest tumour cross-section and (ii) the slices that contained the craniocaudal or mediolateral ends of the tumour (in pancreatoduodenectomy or distal pancreatectomy specimens, respectively). In order to detect possible tumour extension in the craniocaudal or mediolateral direction that was invisible to naked-eye inspection, the slice (or multiple slices in cases with an unclear tumour boundary) flanking the presumed ends of the tumour were also embedded (Figure 1 and Figure 2). Embedding of the specimen slices was performed either by using whole mount blocks or by dividing the specimen slices by rectilinear cuts into tissue samples of standard block size such that the entire tumour bed could be reconstructed during microscopic examination.

For *microscopic measurement* of the tumour dimensions, the invasive front of the tumour was annotated under the microscope in the tissue section(s) representing the specimen slice that was identified as containing the tumour at its largest expanse. The largest tumour dimension, referred to as “2D-micro”, was measured in that tissue section. For evaluation of the largest tumour dimension across specimen slices, the number of specimen slices with microscopically detectable primary tumour growth was recorded and used for the calculation of this tumour dimension (hereafter referred to as “3D-micro”), as outlined above.

All other pathology data collected for this study (N-stage; lymphatic, vascular, perineural invasion; resection margin status) were assessed in line with international guidelines [6].

### 2.4. Statistical Analysis

Descriptive statistics regarding the clinicopathological features of the study cohort are presented as numbers and percentages, both for the entire series and separately for cancers in the pancreatic head and body/tail. The measured tumour dimensions are presented both as means with standard deviations and as medians with interquartile range. Moreover, the overall range for each tumour dimension is presented. Group-wise comparisons between tumours in the pancreatic head and body/tail were performed with independent sample t-tests, while paired comparisons between the different measurement approaches were assessed with paired-samples t-tests. The N-stage distribution for tumours in each T-stage group was compared between the different measurement approaches with Mann–Whitney U tests. A two-sided *p*-value of <0.05 was considered significant. Statistical Package for the Social Sciences, Version 26 (SPSS Inc., Hong Kong) was used for all analyses.

## 3. Results

### 3.1. Study Cohort

A total of 315 eligible cases were included in the study cohort, of which 231 tumours were located in the head of the pancreas and 84 in the body/tail. Pathology data for the entire cohort are presented in Table 1. 

For the purpose of the analysis, the four tumours that were removed by total pancreatectomy were considered as pancreatic head cancers, because these tumours were located mainly in the pancreatic head, with limited extension into the so-called pancreatic neck, that is, the transition to the pancreatic body.

### 3.2. Tumour Size

The results of tumour size assessment macroscopically in the plane of slicing (2D-macro) and across specimen slices (3D-macro) and following microscopic corroboration of both measurements (2D-micro, 3D-micro) are shown in Table 2. Results are stated collectively for the entire series and separately for cancers located in the pancreatic head or body/tail. 

Comparison between both tumour locations revealed that the tumour size measured in the third dimension, that is, across specimen slices, was significantly larger for tumours in the body/tail than for cancers in the head region (*p* < 0.001), both following macroscopic measurement and microscopic corroboration. In contrast, there was no statistically significant difference between locations for the tumour size measured in the plane of slicing.

### 3.3. Differences in Tumour Size Dependent on the Measurement Approach

Because tumour measurement is primarily performed during macroscopic examination, it was first investigated if, and in how far, macroscopic measurement also in the plane perpendicular to the plane of serial slicing resulted in a different tumour size. Furthermore, the impact of microscopic corroboration on tumour size was analysed. The results of comparison of tumour sizes obtained by the different approaches are shown in Table 3.

#### 3.3.1. Impact of Measurement in the Third Dimension (2D-Macro vs. 3D-Macro)

**Figure 3:**
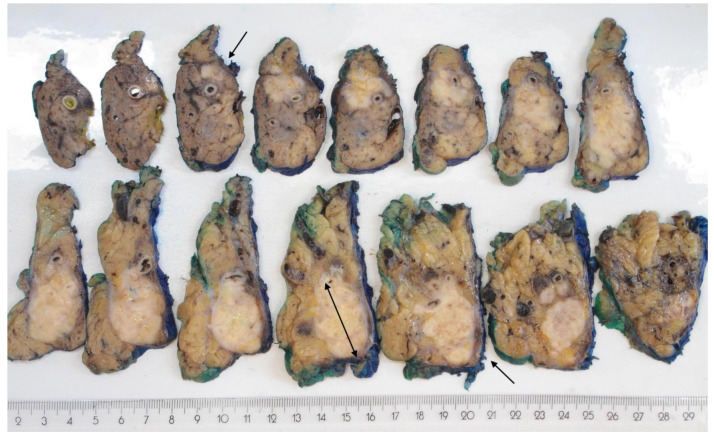
Oblong shape of pancreatic cancer in the body/tail. The 15 sagittal slices of a distal pancreatectomy specimen are laid out in sequential order from upper left to lower right. Tumour tissue is visible from slice 3 (arrow) and extends through multiple subsequent slices. However, it is not clear which of the slices through the pancreatic tail contain tumour or just atrophic tissue. Microscopy revealed that only the two last slices were clear of tumour. The total length of the tumour (from slice 3 to 13, arrows; slice thickness: 4 mm) is 44 mm, which is considerably larger than the maximum dimension in the sagittal plane (34 mm, measured in slice 12) and corresponds with stage T3, not T2.

Macroscopic measurement in the plane perpendicular to the plane of slicing (i.e., across specimen slices; 3D-macro) resulted in tumour sizes that were significantly different from those measured in the plane of slicing (i.e., on a specimen slice; 2D-macro; *p* = 0.003 head, *p* < 0.001 body/tail). The difference in size was particularly large for cancers in the pancreatic body/tail, with a mean size difference of 17 mm. The relative change in size, that is, the difference in size between both measurements divided by the tumour size obtained by 2D macroscopic measurement, was 61%. In 69 (82%) of these cases, the 3D-macro size was *larger* than the 2D-macro size, the mean difference between both sizes being 19 mm (range: 1–73 mm), representing a mean relative change in size of 72% (range: 4–280%). Only in a single case, the tumour size measured in both planes was identical, whereas in 14 (17%) of the body/tail cancers, the 3D-macro size was *smaller* than the one measured in 2D (mean relative change in size: −15%; range: −5% to −50%). As illustrated in Figure 3, the size of the tumour along the length of the pancreatic body/tail (3D-macro) can exceed considerably the cross-sectional size (2D-macro). The study series included one pancreatic cancer in the body/tail that stood out in this respect, because it was considerably wider than it was long (110 mm vs. 55 mm). Interestingly, this outlier was diagnosed as an undifferentiated carcinoma with osteoclast-like giant cells, and it contained a large blood-filled cavity, which was the reason for the unusual shape and exceptionally large cross-sectional size of the tumour.

Statistically significant differences between 2D-macro and 3D-macro sizes were also observed in pancreatic head cancers, although the change in size was overall smaller (mean size difference: 6 mm; median relative change in size: 22%) than in tumours in the body/tail. In 117 (51%) cases, the 3D-macro size was larger than the one obtained by 2D-macro measurement, the mean difference in size being 6 mm (mean relative change in size: 22%). Tumour size was smaller in the 3D plane in 92 (40%) cases, and in 22 (9%) cases, tumour size was identical in both planes.

#### 3.3.2. Impact of Microscopic Corroboration

**Figure 4:**
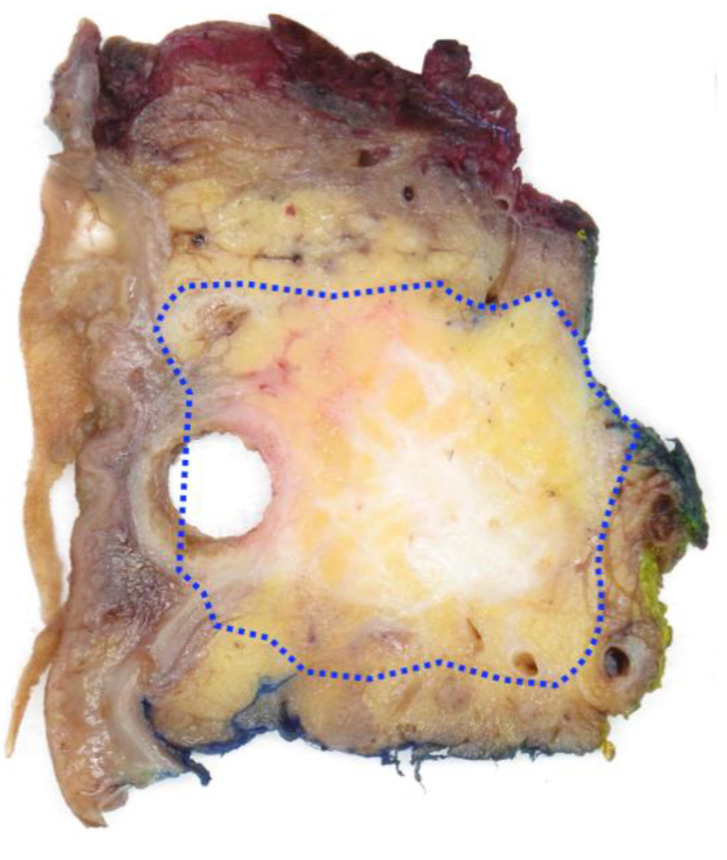
Microscopic corroboration of tumour size. This axial slice from a pancreatoduodenectomy specimen with a largely dilated common bile duct (metal stent removed) shows an ill-defined tumour. The true tumour border, as identified by microscopic examination of this tissue slice, is indicated on the macroscopic image (dotted line) and includes parts of pancreatic tissue that on naked-eye inspection appear clear of tumour. Consequently, the tumour size based on microscopic corroboration (2D-micro) exceeds the one assessed by naked-eye inspection (2D-macro).

For cancers in the pancreatic head, microscopic corroboration of the macroscopic measurement either on a specimen slice (2D) or across axial slices (3D) resulted in significantly different sizes (*p* < 0.001; Table 3). The mean difference in size (and mean relative change in size) introduced by microscopic measurement was 4 mm (17%) or 5 mm (19%), respectively. Microscopic corroboration revealed mainly underestimation of the tumour size that was measured during macroscopic examination (Figure 4). Size *underestimation* by 2D- and 3D-macroscopic measurement was both common (in 136 (59%) and 119 (52%) of the cases, respectively) and substantial (24% and 33% mean relative change in size, respectively), with up to 227% and 183% size change in individual cases, respectively. Size *overestimation* affected fewer cases (59 (25%) by 2D-macro, 35 (15%) by 3D-macro) to a lesser degree (mean relative change in size: −13% and −14%, respectively). 

For cancers in the pancreatic body/tail, microscopic corroboration of sizes in either plane did not reveal significantly different tumour sizes, although especially underestimation of size measured macroscopically across specimen slices was considerable (25% mean relative change in size, range 2-100%) and occurred in 37 (44%) cases.

#### 3.3.3. Impact of Measurement in the Third Dimension Combined with Microscopic Corroboration (2D-Macro vs. Combined 2D-3D-Micro)

**Figure 5:**
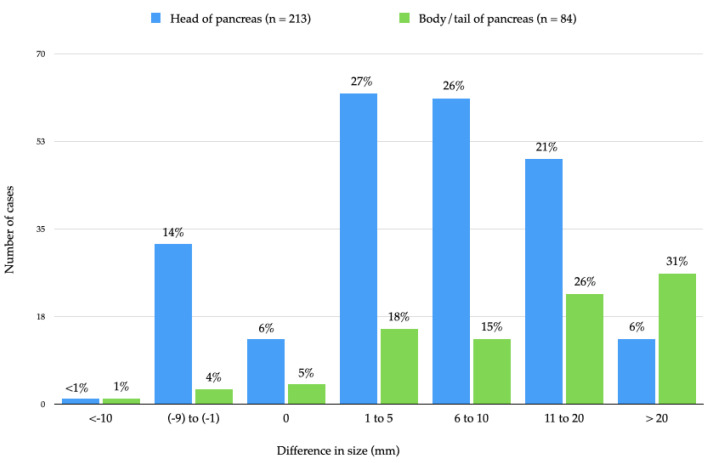
Difference in largest tumour size measured macroscopically in two dimensions (so-called simple approach) versus in three dimensions with microscopic corroboration (so-called comprehensive approach). The graph shows the number of pancreatic head and body/tail cancers (and percentages, indicated at the top of each bar) for the range of differences in size that were observed between both measurement approaches. Negative values indicate that the size obtained by the comprehensive approach was smaller than the one based on the simple approach.

In only 17 (5%) of all the cases, identical maximum tumour sizes were obtained by both a simple procedure, which was based exclusively on macroscopic measurement on a specimen slice (2D-macro), and a comprehensive approach, which consisted of measuring also across specimen slices with microscopic corroboration of both macroscopic sizes (combined 2D-3D-micro). The difference in tumour size based on either approach was statistically significant (*p* < 0.001) irrespective of tumour site (Table 3). In the vast majority of cancers—185 (80%) in the pancreatic head and 76 (90%) in the body/tail—comprehensive measurement revealed a *larger* tumour size than the one obtained by 2D-macroscopic measurement only (Figure 5). Underestimation of the maximum tumour size based exclusively on 2D-macro measurement was not only frequent but also substantial: in 62 (27%) of the cancers in the pancreatic head and 48 (57%) of the tumours in the body/tail, underestimation of size exceeded 10 mm. For pancreatic head cancers, the impact of the comprehensive approach (mean relative change in size: 28%) was larger than that of either the addition of microscopic corroboration of the 2D-macro measurement (mean relative change in size: 17%) or of macroscopic measurement across specimen slices (mean relative change in size: 22%). For cancers in the body/tail, the comprehensive approach resulted in a substantially different tumour size (mean relative change in size: 58%). However, the impact was not significantly larger than that from measurement across specimen slices (mean relative change in size: 61%). 

### 3.4. Impact of the Approach to Tumour Size Measurement on T-Stage Assignment and Distribution

#### 3.4.1. T-Stage Assignment 

For each cancer, T-stage was assigned based on the largest tumour size that was obtained by each of the various measurement approaches. Any difference in T-stage compared to the T-stage based on the simple approach, that is, the macroscopic measurement on a specimen slice (2D-macro), was recorded separately for cancers in the pancreatic head and body/tail (Table 4, Supplementary Table S1). 

When comparing measurement on a specimen slice without versus with microscopic corroboration (2D-macro versus 2D-micro), 57 (18%) cases in the entire study series changed T-category, with a shift affecting 19 of 38 (50%) cancers categorised as T1 based on 2D-macro assessment, 21 of 231 (9%) of those deemed to be T2 and 17 of 46 (37%) cancers that were categorised as T3. 

In 115 (37%) cases, T-stage assignment differed depending on whether tumour size was measured macroscopically in a single plane (2D-macro) or in two planes (3D-macro). This change in T-stage affected 22 of 38 (58%) of the cancers that were categorised as T1 based on 2D-macro assessment, 72 of 231 (31%) of the tumours deemed to be T2 and 20 of 46 (43%) cancers that were categorised as T3. 

Comparison between the simple approach (2D-macro) and the comprehensive approach based on measurement in two planes and microscopic corroboration (2D-3D-micro) revealed an even larger shift in T-stage, namely in 142 of all cases (45%). This change in T-stage affected the vast majority of cancers deemed to be T1 based on the 2D-macro approach (33 of 38, 87%) and more than a third (87 of 231, 38%) and nearly half (22 of 46, 48%) of the tumours in the categories T2 and T3, respectively. In the vast majority of cases with a change in T-stage, the T-stage shifted up (117 of 142, 82%), while in a smaller proportion (25 of 142, 18%), there was a down-shift. The shift was mainly by one T-stage, but in eight cancers (6% of all cases with altered T-stage) the shift was across two T-stages (T1 to T3 in six cases, T3 to T1 in two cases). The single case in the entire series that was assigned to stage T1b based on 2D-macro measurement shifted to T1c, that is, it remained within the T1-category and was therefore not regarded as a change in T-stage. T-stage changed somewhat more frequently for cancers in the pancreatic body/tail than for those located in the pancreatic head (40 of 84 (48%) versus 102 of 213 (44%), respectively). Otherwise, the distribution of changes in the three T-categories was similar irrespective of tumour site.

#### 3.4.2. T-Stage Distribution

The above-described shifts in T-stage resulted in a considerable change in T-stage distribution (Table 5). Tumour size measurement limited to the plane of slicing, without or with microscopic corroboration (2D-macro or 2D-micro), led to a higher proportion of T1-stage cancers (38 (12%) or 27 (9%), respectively) than when using the comprehensive approach (2D-3D-micro), according to which only 10 (3%) cancers were T1. While the proportion of cases assigned to stage T2 also decreased (from 213 (73%) or 245 (78%) to 191 (61%)), the percentage of cases in the T3-category more than doubled when using the comprehensive approach (from 46 (15%) or 43 (14%) to 114 (36%)). Similar changes in T-stage distribution were observed when analysing separately cancers in the pancreatic head or body/tail.

Macroscopic measurement in the plane across specimen slices (3D-macro) introduced changes in T-stage distribution mainly for cancers in the body/tail, where it resulted in a T-stage distribution that was very similar to the one based on the comprehensive approach (T1: 8%, T2: 38%, T3: 54%). In contrast, for cancers in the pancreatic head, T-stage distribution differed little from that based on 2D-macro measurement (T1: 13%, T2: 72%, T3: 15%).

Comparison with the T-stage distribution reported by other series shows that these are similar to the distribution based on the simple approach (2D-macro) but different from the one based on the comprehensive approach (2D-3D-micro; Supplementary Table S2).

Finally, the N-stage distribution (N0, N1, N2) was compared between tumours assigned to stage T1, T2 or T3 based on the simple versus the comprehensive approach (Supplementary Table S3). Based on the comprehensive approach, the proportion of T1 cancers without lymph node metastasis (N0) was higher than for T1 tumours according to the simple approach, and none of the tumours were N2 (vs. 24% of the T1 tumours according to the simple approach; *p* = 0.03). Similar differences in N-stage were observed for T2 tumours (*p* = 0.03), whereas differences were not significant for T3 tumours.

## 4. Discussion

Tumour size is a key oncological data item, because it reflects the primary tumour burden. As for multiple other cancer entities, tumour size is the defining criterion of the T-stage (T1–T3) for ductal adenocarcinoma in the pancreas. The clinical interest in and importance attributed to the TNM system is reflected by the considerable number of studies that soon after the release of the eighth edition of the UICC/AJCC classification system validated the new T- (and N-) staging criteria in European, Asian and US data sets [15,16,17,18,19,20,21,22,23]. While tumour size was the critical data item on which these studies were based, none actually described exactly the method that was used to measure the size of the tumour. Several of the studies mentioned that size was measured during macroscopic examination [18,22,23], while others stated that—at least in part of the cases—the macroscopic size measurement was corroborated microscopically [15,16]. Microscopic corroboration is recommended by national and international pathology guidelines because of the generally poor macroscopic delineation of pancreatic cancer [5,6,7,8,9]. The latter results mainly from the highly dispersed growth of the cancer cells, which occurs especially in the tumour periphery and therefore hampers appreciation of the full width of the tumour by naked-eye inspection [10]. 

This study aimed at investigating whether and to which extent different ways of measuring the tumour resulted in different tumour sizes and in how far this divergence affected T-stage.

Most commonly, pathologists assess tumour size during macroscopic examination of the surgical specimen. Measurement is usually performed only in one plane, namely in the plane of specimen slicing. Following identification of the specimen slice in which the tumour is at its largest expanse, the tumour size is measured on this slice in *two* dimensions, that is, the dimension in which the tumour size is largest and the perpendicular dimension (which is of no further interest to this study, as it is de facto not the largest tumour size). In contrast to this simple procedure, a comprehensive approach includes, in addition, (i) measurement in a *third* dimension, namely in the plane that is perpendicular to the plane of specimen slicing (that is, across specimen slices) and (ii) microscopic corroboration of both sizes that were obtained by macroscopic measurement in both planes (Figure 1 and Figure 2).

To compare the results of the various approaches to tumour size assessment, a set of four sizes were systematically measured in a series of 315 consecutive, surgically resected, treatment-naïve pancreatic cancers. For each case, the following four sizes were recorded: the maximum tumour size measured (i) macroscopically on a specimen slice (referred to as 2D-macro) and (ii) across specimen slices (3D-macro) as well as (iii, iv) the size in either plane obtained by microscopic corroboration (2D-micro, 3D-micro). 

Overall, the study findings reveal that measurement in two planes and microscopic corroboration results in considerable size differences as compared to exclusive macroscopic measurement in a single plane. 

Macroscopic measurement of tumour size both in the plane of sectioning or perpendicular to it, i.e., across specimen slices, resulted in significantly different tumour sizes compared to macroscopic measurement only in the plane of slicing (*p* = 0.003 head, *p* < 0.001 body/tail). The size discrepancy was particularly large for tumours in the pancreatic body/tail: in 82% of cases, measurement limited to the plane of sectioning resulted in underestimation of tumour size by a mean of 19 mm or a 72% relative change in size. This marked difference in size between cross-sectional and longitudinal dimensions is explained by the fact that pancreatic cancers in the body/tail often have an oblong shape, as they grow extensively along the longitudinal axis of the organ without causing cross-sectional expansion to a similar extent (Figure 3). 

As recommended by (inter-)national guidelines, it is common practice to corroborate by microscopic examination the tumour size that is measured macroscopically. This is usually carried out only for the measurement taken on a specimen slice. The study shows that for pancreatic head cancers, tumour size was under- or overestimated in 59% or 25% of cases, respectively, if macroscopic measurement was not corroborated microscopically (mean relative change in size: 24% or 13%, respectively; *p* < 0.001). For cancers in the body/tail, size differences were not statistically significant, which is not surprising considering that the majority of these cancers already occupy the entire width of the pancreatic body/tail with—as outlined above—limited expansion of the latter. 

Similar observations were made when comparing measurements across specimen slices with or without microscopic corroboration. Significant size differences were observed for pancreatic head cancers (*p* < 0.001), with size underestimation in 52% (mean relative change in size 33%) if macroscopic measurement was not corroborated microscopically. For cancers in the body/tail, size differences were not statistically significant. However, in individual cases, large size discrepancies—overestimations of up to 29 mm or underestimations of up to 60 mm—were recorded. The reason for these large differences is the fact that pancreatic tissue upstream from the tumour is often severely affected by atrophy and fibrosis, which renders macroscopic distinction from the cancer difficult (Figure 3).

Finally, results from the most commonly used, simple approach of exclusive macroscopic measurement on a specimen slice were compared with those obtained by a comprehensive approach that combines macroscopic assessment in two planes *and* microscopic corroboration of both measurements. In only 5% of all cases, the largest tumour size was the same by either approach. Tumour sizes were significantly different irrespective of tumour site (*p* < 0.001). In 83% of all cases, the comprehensive approach revealed a larger tumour size, which in 35% exceeded by more than 10 mm the size obtained by the simple approach (Figure 5).

Because tumour size was found to differ significantly depending on the approach to measurement, it was further investigated if, and to which extent, this affected the T-stage in each case. While microscopic corroboration of tumour size measured in a specimen slice affected the T-stage in only 18% of cases, macroscopic measurement in the plane across specimen slices caused a T-shift in 37%. When comparing the simple with the comprehensive approach, the T-stage changed in 45% of cancers, in 82% of these to a higher T-stage. These shifts had a knock-on effect on T-stage distribution. Comparing the simple to the comprehensive approach, the proportion of T1 tumours was reduced from 12% to a dwindling 3%, the group of T2 tumours was also reduced (from 73% to 62%), while T3 tumours more than doubled (from 15% to 36%). Of note, T1 and T2 tumours, according to the comprehensive approach, were associated with a lower N-stage than those deemed to be T1 or T2 according to the simple approach. This observation indicates that T1 and T2 tumours identified by the comprehensive approach truly represent an earlier cancer stage with, accordingly, a lower rate of lymph node metastasis.

Taken together, the study demonstrates that various measurement approaches lead to significant differences in tumour size and consequently a different pT assignment in a considerable number of cases. The current most commonly used approach, which is based only on macroscopic measurement in the plane of specimen slicing, results in a different, incorrect T-stage assignment in 45% of pancreatic cancers as compared to the comprehensive approach.

To the best of our knowledge, this study is the first to show that different approaches to tumour measurement result in significantly different tumour sizes and consequently assignment to different T-stages. The study is also the first to reveal that identification of the largest tumour size is dependent on two key aspects of the pathology examination method: measurement in two planes, i.e., on a specimen slice and across slices and microscopic corroboration. While the latter is relevant irrespective of the tumour site, the former affects particularly (but not exclusively) the size assessment of tumours in the body/tail, resulting in a mean relative change in size of no less than 61%. Furthermore, the results show that because size discrepancies are both common *and* substantial, they lead to a shift in T-stage in 45% of tumours, if the comprehensive approach is used instead of the simple approach. As such, the assumption that measurement of tumour size is accurate and reproducible—and results in correct T-staging—only applies if the comprehensive method is used systematically. 

The findings in this study have two important implications. First, given the current divergence in practice related to tumour size measurement, tumour size and T-stage assignment may not be comparable between institutions. While most guidelines acknowledge that microscopic corroboration is important [5,7,8,9], published studies show that this recommendation is not uniformly put into practice [16,17,18,20,22,23]. Moreover, because the lack of three-dimensional tumour measurement results in inaccurate tumour size and T-stage in a significant proportion of cases—95% and 45% in this series—bias may be introduced in multicentre clinical trials and tumour registries. Furthermore, the correlation with patient outcome or other parameters that are tested for predictive or prognostic significance may be affected. In that respect, it is interesting to note that the T-stage distribution differs remarkably between the studies that validated the eighth edition of the AJCC/UICC TNM classification system (T1: 12-20%, T2: 55-69%, T3:17–28%; Table S2). These published data are similar to the T-stage distribution observed in the current study based on the simple approach but differ considerably from the stage distribution based on the comprehensive approach, the latter resulting in a remarkably smaller T1-category (3%) and larger T3-category (36%). 

Since the ease of practice is an important factor to consider when deciding on which approach to follow, one could argue that universal adherence to the currently more commonly used approach, namely macroscopic measurement in the plane of specimen slicing without microscopic corroboration, would ensure comparability of data. However, while this would indeed improve reproducibility, it would not address the fact that—as outlined above—in a large proportion of cases both tumour size and T-stage are incorrect, with either under- or overestimation of a highly variable degree. 

A second implication of this study is that (inter-)national data sets for pancreatic cancer should not only recommend tumour size measurement in three dimensions but also provide practical guidance as to how this should be carried out, an important aspect that currently is lacking. Macroscopic measurement of the tumour dimension across specimen slices involves more detailed recording during macroscopic examination, and microscopic corroboration of this tumour dimension requires additional tissue sampling. In the experience of the authors, this required approximately 5 min of additional work. While more demanding, the comprehensive approach can be easily included in the standard operating protocol for specimen grossing (used by either pathologists or technical staff) and microscopic assessment. 

The study has several limitations. First, the analysis is based on a single-institution cohort, which may have introduced bias. However, because clinical, surgical and pathology methods and decisions were standardised according to international guidelines [6,24], the bias is likely limited. Second, for pancreatoduodenectomy specimens, grossing was based on axial slicing, not bivalving as it is practised in some pathology departments [25]. However, the different specimen dissection techniques are expected to have little if any impact when it comes to the measurement of tumour size, because the exact orientation of the dissection plane (axial versus along the main pancreatic and common bile ducts in the case of bivalving) is of little relevance as long as measurement is carried out in two perpendicular planes, which is the case for both techniques. More relevant is the slice thickness, as this determines the accuracy of both size measurement across specimen slices and the identification of the specimen slice with the largest tumour expanse. Slice thickness is not often mentioned in published studies, but when stated it lies around 3-5 mm [26]. In the current study, tumour size was measured with 1.5 mm increments across specimen slices. Dissection of distal pancreatectomy specimens by serial sagittal slicing is almost universally used, and it is the preferred method for international multicentre trials [27]. As specimen grossing is usually undertaken following fixation, formalin-induced tissue shrinkage is unlikely to have a significant impact on the reproducibility of tumour size measurement.

A third limitation is the relatively small size of the cohort (n = 315). However, as discussed above, the mean and median tumour sizes observed in the current study for two-dimensional measurement are highly comparable with data from other studies [15,16,17,18,19,20,21,22,23], indicating that the impact of the size of the study series is likely small. The exclusion of specimens from neoadjuvantly treated patients represents a further limitation, especially as the proportion of the latter is rapidly increasing. However, the challenges met when measuring the size of residual cancer following neoadjuvant treatment are of an entirely different nature and have not been addressed as yet, making exclusion of this patient group unavoidable [28]. A further shortcoming of the study lies in the lack of a comparison with imaging-based tumour sizes, which were shown to underestimate tumour size measured at a microscopic level [29]. 

Last but not least, the study did not investigate the correlation between tumour size and/or T-stage and patient survival. The reason for this omission is the fact that survival is determined by multiple factors other than tumour size and T-stage, in particular N-status and, for instance, adjuvant treatment. Indeed, the intention of the TNM system is in the first instance to allow unambiguous and precise communication of the tumour burden between multidisciplinary user groups, a goal to which the current study findings have contributed [30]. 

The strengths of the study lie in the prospective data collection, the fact that the study series is recent and covers a short period of time (2015–2020) and the use of a detailed, fully standardised pathology examination protocol. A further strength is the extensive annotation of the study series, including macroscopic photo documentation of all cases, which enables review not only of the histology but also of the macroscopic findings. 

## 5. Conclusions

While tumour size measurement is generally considered accurate and reproducible, the study results show that different measurement approaches result in significantly different maximum tumour sizes and, consequently, significantly different T-stage assignment. Especially measurement in the plane perpendicular to the plane of specimen slicing, but also microscopic corroboration of the macroscopic measurements, has a significant impact on the resulting tumour size and T-stage assignment. As long as pathology practice varies, tumour size and T-stage may be neither accurate nor reproducible. This puts at risk the meaningfulness of comparing stage-related outcomes between institutions, to the detriment of patient management, clinical research and cancer registries. Even if, in the not-too-far future, artificial-intelligence-assisted tumour size measurement may overcome current obstacles, study cohorts with correctly measured pancreatic cancers will be invaluable for establishing a robust ground truth [31]. 

## Figures and Tables

**Table 1:** Clinicopathological features.

	Entire Series (*n* = 315) (%)	Head of Pancreas (*n* = 231) (%)	Body/Tail of Pancreas (*n* = 84) (%)
**Specimen type**			
Pancreatoduodenectomy			
Classical	80 (25)	80 (35)	0 (0)
Pylorus-preserving	147 (47)	147 (64)	0 (0)
Distal pancreatectomy	84 (27)	0 (0)	84 (100)
Total pancreatectomy	4 (0.01)	4 (0.02)	0 (0)
T-stage *			
T1c	10 (3)	5 (2)	5 (6)
T2	190 (60)	155 (67)	35 (42)
T3	115 (37)	71 (31)	44 (52)
**N-stage**			
N0	32 (10)	18 (8)	14 (17)
N1	115 (37)	79 (34)	36 (43)
N2	168 (53)	134 (58)	34 (40)
**Lymphatic invasion**			
L0	22 (7)	11 (5)	11 (13)
L1	293 (93)	220 (95)	73 (87)
**Vascular invasion**			
V0	93 (30)	69 (30)	24 (29)
V1	199 (63)	161 (69)	38 (45)
V2	23 (7)	1 (1)	22 (26)
**Perineural invasion**			
Pn0	22 (7)	9 (4)	13 (15)
Pn1	293 (93)	222 (96)	71 (85)
**Resection margin status**			
R0	52 (17)	37 (16)	15 (18)
R1	263 (83)	194 (84)	69 (82)

* Based on 2D-3D-micro measurement.

**Table 2:** Tumour size obtained by various measurement approaches.

Tumour Size (mm)	Entire Series (*n* = 315)	Head of Pancreas (*n* = 231)	Body/Tail of Pancreas (*n* = 84)
**2D-macro**			
Mean (SD)	30 (10)	30 (8)	31 (15)
Median (IQR)	30 (23–35)	30 (27–40)	27 (21–36)
Range	9–110	11–51	9–110
**2D-micro**			
Mean (SD)	32 (10)	32 (7)	31 (15)
Median (IQR)	31 (25–36)	32 (27–36)	29 (21–36)
Range	6–110	14–61	6–110
**3D-macro**			
Mean (SD)	35 (14)	32 (10)	45 (19)
Median (IQR)	32 (25–41)	30 (25–37)	43 (30–55)
Range, mm	12–100	12–61	13–100
**3D-micro**			
Mean (SD)	38 (14)	35 (10)	45 (19)
Median (IQR)	36 (28–45)	35 (28–41)	43 (32–59)
Range	13–100	15–75	13–100

SD: standard deviation; IQR: interquartile range.

**Table 3:** Difference in tumour sizes obtained by various approaches.

Difference in Tumour Size	Head of Pancreas			Body/Tail of Pancreas		
	Absolute (mm)	Relative * %	*p*-Value	Absolute (mm)	Relative * %	*p*-Value
**2D-macro vs. 2D-micro**			<0.001			0.73
Mean (SD)	4 (4)	17 (24)		3 (5)	11 (14)	
Range	−21–25	−50–227		−22–30	−52–60	
**2D-macro vs. 3D-macro**			0.003			<0.001
Mean (SD)	6 (6)	22 (23)		17 (16)	61 (59)	
Range	−27–24	−64–123		−55–73	−50–280	
**3D-macro vs. 3D-micro**			<0.001			0.83
Mean (SD)	5 (6)	19 (26)		7 (10)	17 (20)	
Range, mm	−22–28	−38–183		−60–29	−63–100	
**2D-macro vs. 2D-3D-micro**			<0.001			<0.001
Mean (SD)	7 (8)	28 (40)		16 (15)	58 (57)	
Range	−21–37	−50–308		−15–73	−36–290	

* Relative difference in tumour size measured by approach A and B: $\frac{\left(\mathrm{tumour}\;\mathrm{size}\;\mathrm{approach}\;A)-(\mathrm{tumour}\;\mathrm{size}\;\mathrm{approach}\;B\right)}{\mathrm{tumour}\;\mathrm{size}\;\mathrm{approach}\;A}\times 100$. SD, standard deviation.

**Table 4:** Shift in T-stage assignment when comparing various approaches to tumour size measurement.

Shift in T-Stage	None *n* (%)	Shift to Higher T-Stage *n* (%)	Shift to Lower T-Stage *n* (%)
**2D-macro vs. 2D micro**			
Head	186 (80)	30 (13)	15 (7)
Body/tail	72 (86)	3 (3)	9 (11)
**2D-macro vs. 3D-macro**			
Head	161 (70)	36 (15)	34 (15)
Body/tail	39 (46)	39 (46)	6 (8)
**2D-macro vs. 2D-3D-micro**			
Head	129 (56)	80 (35)	22 (9)
Body/tail	44 (52)	37 (44)	3 (4)

**Table 5:** Distribution of T-stage based on various approaches to tumour size measurement.

Head of Pancreas (*n* = 231)	2D-Macro *n* (%)	2D-Micro *n* (%)	2D-3D-Micro *n* (%)
T1	26 (11)	10 (4)	5 (2)
T2	176 (76)	193 (84)	156 (68)
T3	29 (13)	28 (12)	70 (30)
**Body/tail of pancreas** **(*n* = 84)**			
T1	12 (14)	17 (20)	5 (6)
T2	55 (66)	52 (62)	35 (42)
T3	17 (20)	15 (18)	44 (52)

## Data Availability

Upon request, data will be made available by the corresponding author.

## Supplementary Materials

The following supporting information can be downloaded at: https://www.mdpi.com/article/10.3390/cancers14102471/s1, Table S1: Change in T-stage assignment between different approaches to tumour size measurement; Table S2: T-stage distribution in pancreatic cancer series; Table S3: N-stage distribution for T-stages according to different approaches to tumour size measurement.
