# Supplementary material for: Tumour Size and T-Stage in Pancreatic Cancer Resection Specimens Depend on the Pathology Examination Approach
Source: Cancers (Basel). 2022 May 17;14(10):2471. doi: 10.3390/cancers14102471 (PMC9139767; doi:10.3390/cancers14102471)
Supplement: Supplementary file 1 [file cancers-14-02471-s001.zip › cancers-1684018-supplementary.pdf]

cancers MDPI

Supplementary Materials

Tumour Size and T-Stage in Pancreatic Cancer Resection Specimens Depend on the Pathology Examination Approach

Table S1. Change in T-stage assignment when comparing the simple approach (2D-macro) with the three more elaborate approaches to tumour size measurement.

	2D-Macro vs. 2D Micro		2D-Macro vs. 3D-Macro		2D-Macro vs. 2D-3D-Micro	
	**Head**	**Body/Tail**	**Head**	**Body/Tail**	**Head**	**Body/Tail**
**T1**	**(*n* =26) ***	**(*n* = 12) ***				
No changes	8 (31)	11 (92)	12 (46)	4 (33)	1 (4)	4 (33)
T1 → T2	18 (69)	1 (8)	13 (50)	7 (58)	19 (73)	8 (67)
T1 → T3	0 (0)	0 (0)	1 (4)	1 (9)	6 (23)	0 (0)
**T2**	**(*n* = 176) ***	**(*n* = 55) ***				
No changes	162 (92)	48 (87)	137 (78)	22 (40)	119 (68)	25 (45)
T1 → T2	12 (7)	12 (7)	22 (12)	30 (55)	55 (31)	29 (53)
T1 → T3	2 (1)	5 (9)	1 (4)	3 (5)	2 (1)	1 (2)
**T3**	**(*n* = 29) ***	**(*n* = 17) ***				
No changes	16 (55)	13 (76)	12 (41)	14 (82)	9 (31)	15 (88)
T1 → T2	13 (45)	3 (18)	15 (52)	3 (18)	18 (62)	2 (12)
T1 → T3	0 (0)	1 (6)	2 (7)	0 (0)	2 (7)	0 (0)

* Number of tumours assigned to the T-category based on 2D-macro assessment.

Table S2. T-stage distribution in pancreatic cancer series.

Study	% T1	% T2	% T3
**Current**			
• 2D-macro	12	73	15
• 2D-3D-micro	3	61	36
**Saka 2016** [22]			
• Institutional series	13	64	23
• SEER series	17	55	28
• “Ordinary cases”	20	56	24
**Shin 2019** [18]	13.1	66.5	20.5
**Kwon 2018** [20]	19.6	63.8	16.6
**Park 2019** [17]	14.5	65.5	20.0
**Schlitter 2017** [23]			
• Berlin series	12.0	69.3	18.7
• Munich series	13.5	65.3	21.2
**Kamarajah 2017** [19]	17	58	25

Table S3. *n*-stage distribution for T-stages according to different approaches to tumour size measurement.

	N0<br>*n* (%)	N1<br>*n* (%)	N2<br>*n* (%)	*p*-Value
**T1**				0.03
• 2D-macro	9 (24)	19 (52)	9 (24)	
• 2D-3D-micro	5 (56)	4 (44)	0 (0)	
**T2**				0.03
• 2D-macro	21 (9)	78 (34)	132 (57)	
• 2D-3D-micro	22 (12)	81 (42)	88 (46)	
**T3**				0.20
• 2D-macro	1 (2)	18 (39)	27 (59)	
• 2D-3D-micro	4 (4)	30 (26)	80 (70)	
